# Sensitivity study of an automated system for daily patient QA using EPID exit dose images

**DOI:** 10.1002/acm2.12303

**Published:** 2018-03-06

**Authors:** Audrey H. Zhuang, Arthur J. Olch

**Affiliations:** ^1^ Department of Radiation Oncology University of Southern California Los Angeles CA USA; ^2^ Department of Radiation Oncology University of Southern California Children's Hospital Los Angeles Los Angeles CA USA

**Keywords:** automated treatment delivery verification, *in vivo* dosimetry, patient safety, transit dosimetry

## Abstract

The dosimetric consequences of errors in patient setup or beam delivery and anatomical changes are not readily known. A new product, PerFRACTION (Sun Nuclear Corporation), is designed to identify these errors by comparing the exit dose image measured on an electronic portal imaging device (EPID) from each field of each fraction to those from baseline fraction images. This work investigates the sensitivity of PerFRACTION to detect the deviation caused by these errors in a variety of realistic scenarios. Integrated EPID images were acquired in clinical mode and saved in ARIA. PerFRACTION automatically pulled the images into its database and performed the user‐defined comparison. We induced errors of 1 mm and greater in jaw, multileaf collimator (MLC), and couch position, 1° and greater in collimation rotation (patient yaw), 0.5–1.5% in machine output, rail position, and setup errors of 1–2 mm shifts and 0.5–1° roll rotation. The planning techniques included static, intensity modulated radiation therapy (IMRT) and VMAT fields. Rectangular solid water phantom or anthropomorphic head phantom were used in the beam path in the delivery of some fields. PerFRACTION detected position errors of the jaws, MLC, and couch with an accuracy of better than 0.4 mm, and 0.5° for collimator rotation error and detected the machine output error within 0.2%. The rail position error resulted in PerFRACTION detected dose deviations up to 8% and 3% in open field and VMAT field delivery, respectively. PerFRACTION detected induced errors in IMRT fields within 2.2% of the gamma passing rate using an independent conventional analysis. Using an anthropomorphic phantom, setup errors as small as 1 mm and 0.5° were detected. Our work demonstrates that PerFRACTION, using integrated EPID image, is sensitive enough to identify positional, angular, and dosimetric errors.

## 1 INTRODUCTION

Medical physicists perform a wide array of quality assurance (QA) measures in support of all patient treatments as well as those that are patient‐specific prior to the start of treatment. However, once treatment has started, other than weekly chart checks, there are few if any efforts to verify ongoing patient‐specific treatment delivery accuracy. With the advent of complex treatments and tightening target volume margins, image guided treatments are being performed more frequently with the objective of assuring isocenter positional accuracy and reproducible body pose. These efforts, while necessary, are not sufficient to assure that the correct radiation dose is being delivered daily.

The checks that are done prior to the start of the patient's treatment, such as chart and plan checks, and patient‐specific QA will not catch errors caused by patient anatomy changes, patient setup errors, and machine output errors. Patient‐specific intensity modulated radiation therapy (IMRT) QA tests involving 2D Gamma passing rates commonly done prior to start of treatment have been found to be insufficient to verify the actual dose received by the patient.^1^, ^2^

*In vivo* dose verification has been performed to verify delivered dose, typically only during the first fraction using point dose detectors such as diode, thermoluminescent dosimeters and optically stimulated luminescent dosimeters, and metal‐oxide semiconductor field effect transistor.^3^, ^4^, ^5^, ^6^ However, a point dosimeter can easily miss the errors that affect the area outside of the measurement point and can be insensitive to small errors because of placement uncertainty and movement due to patient breathing. It typically requires labor for placement, pretreatment calibration, and posttreatment readout. It also has dependence on some of treatment parameters such as accumulated dose, energy, SSD, field size, linearity, angular orientation, and readout delay, and in general, a point dosimeter has a measurement uncertain up to 3–5%.^6^

The Electronic Portal Image Device (EPID) has the advantage of being integrated into most linear accelerators (linac) and is ready to measure QA plans or patient exit dose during treatment delivery. With submillimeter spatial resolution, and excellent dose measurement accuracy, linearity to dose and dose rate, and capability of collecting the integrated signal or dynamic signal, the EPID has been widely used for machine QA and pretreatment verification such as patient‐specific IMRT verification.^7^, ^8^, ^9^, ^10^, ^11^ Recently many authors have investigated using EPID for *in vivo* dosimetry.^6^, ^12^, ^13^, ^14^ Some authors compared reconstructed EPID‐based 3D dose distribution inside the patient to the original treatment plan,^6^, ^13^, ^14^ and some authors compared the EPID‐measured doses to the predicted doses at the EPID level.^12^ In addition, some authors implemented real time dose delivery verification by comparing EPID‐measured images to calculated model‐generated transit EPID images.^12^ Most of these prior efforts have been manually performed.

PerFRACTION (Sun Nuclear Corporation, Melborne, FL, USA) is a system that automatically monitors the consistency of daily treatment delivery using the EPID. PerFRACTION automatically retrieves the EPID exit dose images from the radiotherapy electronic medical record (EMR) system database after each treatment fraction for each patient monitored by the system. A user‐defined comparison test such as the gamma analysis is performed for each beam and each fraction against a user‐defined baseline fraction. Using the EPID images, PerFRACTION has the potential to identify changes in patient anatomy, patient setup, beam delivery, or couch rail positions that could affect the treatment delivery.^15^ In order for the test results to be meaningful, the accuracy and sensitivity of the system to measure the changes in dose that can occur during treatment must be characterized. In this work, we investigate the sensitivity of PerFRACTION to detect the deviation of induced errors in a variety of realistic scenarios. As far as we can tell, this is the first publication which presents such data.

## 2 MATERIALS AND METHODS

### 2.A PerFRACTION system overview

The system (PerFRACTION version 1) consists of a dedicated server running embedded Microsoft windows, database software, and a web interface for configuration and data analysis. A DICOM file transfer connection is made between PerFRACTION and the user EMR in our case, ARIA (Varian Medical Systems, Palo Alta, CA, USA). An integrated treatment beam image is taken for each field for each treatment. The acquired EPID images are automatically saved in ARIA and PerFRACTION automatically retrieves the images from ARIA using an automated query retrieve process. Images of each field measured by the EPID during the first fraction are normally chosen to be baseline images, and images captured during each subsequent fraction are compared against the baseline images. The process requires minimal effort because PerFRACTION automatically compares new images against baseline images using various user‐defined tests including the Gamma analysis^16^ with user‐defined percent dose difference (DD), distance‐to‐agreement tolerances, dose threshold, and passing rate that is a percentage of pixels passing the criteria. If the percentage of passing points does not meet a preset passing rate (i.e. 95%), PerFRACTION will notify the physicist via email. A web‐based interface can also be used to review results for each field for each fraction. More recent versions of the system can also perform 3D dose calculations using cine images of multileaf collimator (MLC) positions with log file information on monitor units (MU) at each control point.

### 2.B Linear accelerator, MLC, EPID, and acquisition mode

All treatments in this work were delivered on a TrueBeam (Varian Medical System) with a Millennium 120‐leaf MLC using 6 megavoltage (MV) photon beams. An EPID (Varian aS1000 flat panel detector) was used to acquire MV integrated exit dose images. The detector has an area of 40 cm × 30 cm with a matrix of 1024 × 768 pixels, which provides a spatial resolution of 0.39 mm × 0.39 mm. The patient support was the QFIX (QFIX, Avondale, PA) replacement for the standard Varian couch top, which included the Dosemax couch insert and movable rails. Images were saved in the ARIA database in the same way as any patient treatment images. All images were measured at the source to detector distance (SDD) of 150 cm and were not scaled back to 100 cm SDD except for those in the MLC errors with IMRT test (Section 2.C.8) that were analyzed using PerFRACTION version2.

### 2.C Experimental design

To investigate PerFRACTION's sensitivity in detecting various treatment errors, nine experiments were performed, simulating machine errors (jaw position, MLC leaf position, collimator rotation, MUs, and output), couch errors (couch position, and rail position), errors in IMRT fields, and patient setup errors. In each case, the first delivery was done with nominal machine parameters, and the EPID imager was used to measure an integrated image which was defined as a baseline image. Subsequent fractions of errant machine conditions were delivered and EPID‐measured images were compared against the baseline image.

To evaluate PerFRACTION's sensitivity in detecting geometric errors such as jaw position, MLC position, collimator rotation, and couch shift errors as in Section 2.C.1‐3, and 2.C.5, we used the gamma analyses in PerFRACTION, but we suppressed the DD aspect by setting the DD tolerance to zero, so the tolerance for distance‐to‐agreement (DTA) determines if a pixel passes or fails the comparison criteria. We refer to this method as the DTA method in this paper, which is defined as the distance between a point in an image compared to the nearest point with the same dose in another image. If the DTA value is out of tolerance, the gamma value is >1; if the distance is within tolerance, the gamma value is ≤1. PerFRACTION renders the pixel to be orange if the gamma is >1 and renders a pixel to be between green and yellow for gamma between 0 and 1. We first set the DTA tolerance to be greater than the value of an induced error for which we expected no failing pixels. Then, we decreased the DTA tolerance in 0.1 mm increments until failing pixels began to appear in the color map, and recorded the final tolerance value. In all cases, failing pixels only occurred with DTA tolerances less than the induced error. The difference between an actual induced error and the tolerance implies the sensitivity of the PerFRACTION system.

To evaluate PerFRACTION's sensitivity in detecting dosimetric errors such as machine output error as in Section 2.C.4 and 2.C.6‐7, we used the PerFRACTION‐calculated DD method. The DD method is defined as the DD between a point in an image and a point at the same location in a baseline image. If the DD is outside the tolerance, PerFRACTION renders the pixel to be red or blue dependent on if it is too hot or too cold compared to the baseline. We first set the DD tolerance to be slightly above an induced error and noted the presence of blue or red pixels in the dose map. Then we decreased the tolerance in 0.1% increments until blue or red pixels began to appear in the resultant dose map, and recorded the final tolerance value which represented the magnitude of error recognized by PerFRACTION. The difference between an actual induced error and the tolerance implies the sensitivity of the PerFRACTION system.

#### 2.C.1 Jaw position errors

**Figure 1:**
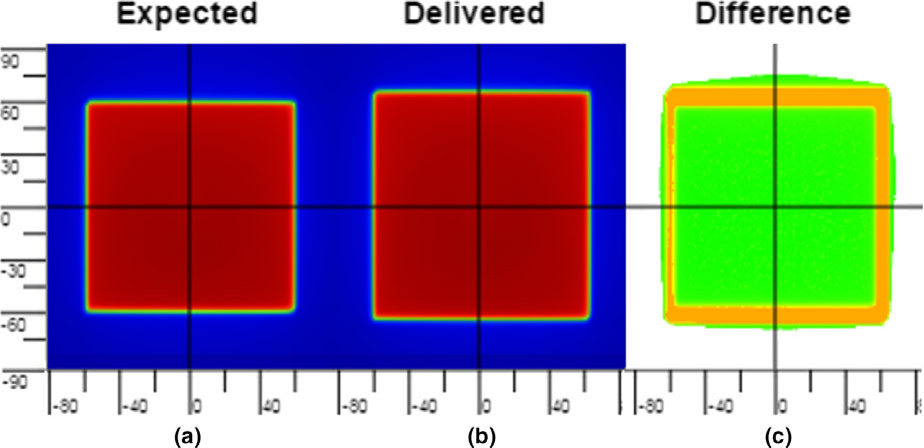
(a) EPID‐measured baseline image. (b) EPID image with induced jaw position errors. (c) Color map of DTA analyses generated by PerFRACTION, in which the area with erroneous jaw position was painted in orange color.

A 10 cm × 10 cm open field was delivered with the gantry at 0° (IEC) and 100 MUs. Then the field was delivered again with X1, X2, Y1, and Y2 jaws increased by 1, 2, 3, or 4 mm from the nominal positions respectively (Fig. 1). No phantom or couch was intersecting the beam. A PerFRACTION‐calculated DTA analyses was used for image comparison.

#### 2.C.2 MLC leaf position errors

**Figure 2:**
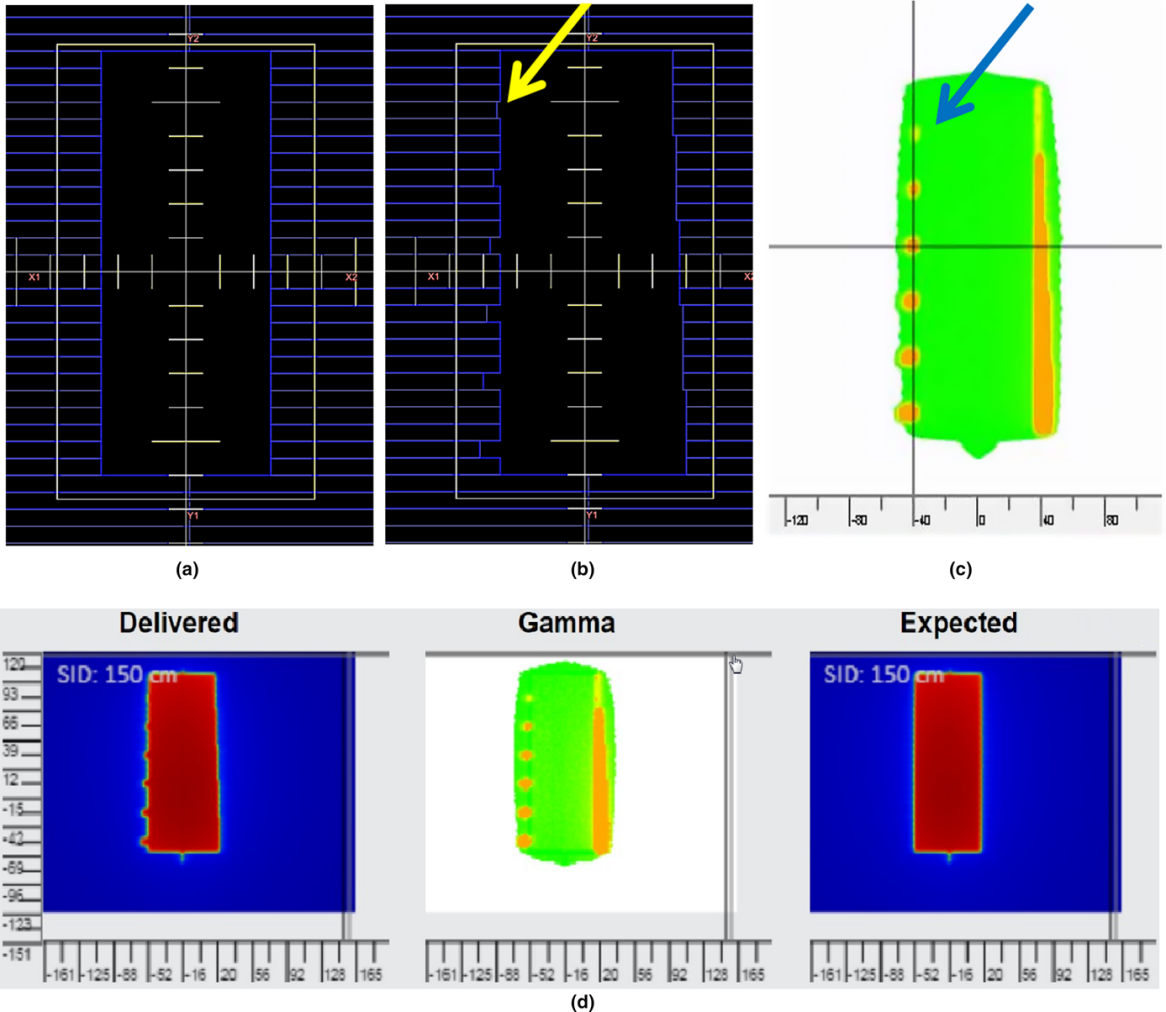
MLC shift error in static fields with and without a phantom. (a) Baseline positions of MLC leaves. (b) MLC leaf positions with induced errors. The yellow arrow points to the area where the leaf was shifted from baseline by 1 mm. (c) Color map of DTA analyses generated by PerFRACTION that displays the area with erroneous leaf positions in orange color. The blue arrow points to the area where the leaf shifted from baseline by 1 mm when the DTA was within 0.4 mm of that nominal value. (d) The MLC leaf position test with 20 cm thick phantom in the beam. Display of EPID image with MLC shifted from baseline (left) and in baseline positions (right), and display of DTA analyses (middle).

A 13 cm × 5 cm MLC‐shaped field with predetermined leaf positions was delivered with gantry at 0° (IEC) and 100 MUs (Fig. 2). Then the field was delivered again with every 4th MLC leaf shifted from nominal positions in the leaf motion direction between 1 and 6 mm on the left bank (as seen on a beam's eye view). The leaves on the right bank were shifted between 1 and 5 mm in groups of five (Fig. 2). This test was repeated with the beam passing through a 20 cm rectangular solid water phantom to determine the effects of scatter on the analysis. PerFRACTION‐calculated DTA analyses were used for image comparison.

#### 2.C.3 Collimator rotation errors

**Figure 3:**
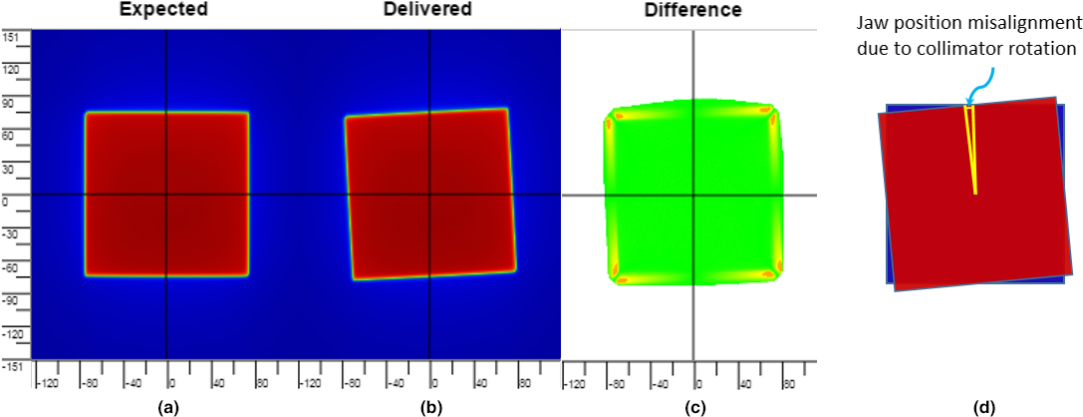
Collimator rotation error test. (a) The EPID‐measured baseline image with a correct collimation angle. (b) Collimator with an induced angular error. (c) Color map of DTA analyses result generated by PerFRACTION, in which the region with collimator position errors was painted in yellow to orange color. (d) Illustration of jaw position misalignment due to collimator rotation.

A 10 cm × 10 cm open field with nominal collimator rotation (i.e. 0°) was delivered with 100 MUs. The gantry at 0° (IEC), and no phantom or couch was intersecting the beam. Then the field was delivered three times with collimator rotated 1, 2, and 3°. A collimator rotation caused an apparent jaw position misalignment (Fig. 3). A rotation of 1° around the center of a 15 cm × 15 cm field (our 10 cm × 10 cm field with a 1.5 magnification factor on the EPID) will cause a jaw position deviation of 1.3 mm which is obtained by multiplying the half length of the field side by the tangent of 1°. The DTA method was used for image comparison to evaluate PerFRACTION's sensitivity in detecting this geometric error.

#### 2.C.4 Machine output error

A 10 cm × 10 cm open field was delivered with 100 MUs and gantry at 0° (IEC) to a 10 cm thick rectangular solid water phantom. Then the field was delivered three times with the MUs changed by 0.5%, 1% and 1.5% relative to the nominal MUs. A PerFRACTION‐calculated DD method was used for error analyses.

#### 2.C.5 Couch position errors

A 10 cm × 10 cm open field was delivered with 100 MUs, gantry at the 0° (IEC) and the couch intersecting the beam. The couch top has carbon fiber strips 5 mm wide, 3 mm thick, and 3 cm apart. Integrated exit dose images were taken on the EPID which clearly shows the strips. Then the field was delivered three times with the couch moved laterally by 1, 2, and 3 mm relative to the nominal position. A PerFRACTION‐calculated DTA method was used for error analyses.

#### 2.C.6 Rail position error with a static field

An 18 cm × 18 cm open field was delivered with the beam passing through the couch and the rails at the out‐most position of the couch. The couch surface was at the level of 100 cm source to surface distance, fully extended and centered. Then the field was delivered with the rails moved to the inner‐most position of the couch. The gantry was at 45° (IEC) and 100 MUs was delivered with each field. A PerFRACTION‐calculated DD method was used for error analyses.

#### 2.C.7 Rail position error with a VMAT field

A 10 cm × 10 cm VMAT field was delivered with the arc passing through the couch and the rails at the outer‐most position. The isocenter was at the couch surface at its center. Then the field was delivered again with the rails moved to the inner‐most position of the couch. 300 MUs was delivered with each arc field. A PerFRACTION‐calculated DD method was used for error analyses.

#### 2.C.8 MLC errors in IMRT fields

Three IMRT fields, which are a part of a plan treating the RTOG H&N phantom, were used to test PerFRACTION's sensitivity in detecting MLC errors in IMRT field delivery. For this test, PerFRACTION v2 was used with the only material difference from v1 being that images taken at an SDD of 150 cm are rescaled back to 100 cm. The MLC errors were induced by changing the fields’ fluence map which, after running the Varian leaf motion calculator program, causes the MLCs to shift from nominal positions. This created three pairs of fields that had a gamma passing rate approximately between 85 and 95% for 2% DDs and 2 mm distance‐to‐agreement. Both the baseline fields and the test fields were delivered with a 20 cm thick rectangular phantom in the beam path (to provide a realistic degree of scatter) with an SSD of 90 cm. PerFRACTION v2 rescaled the EPID images to 100 cm SDD and performed the gamma analyses using the criteria of 2%/2 mm and 10% dose threshold. The same fields were delivered again in the same geometry except that a MapCHECK2 (Sun Nuclear Corporation, Melborne, FL, USA), with 5 mm diode spacing and with the detector plane at 150 cm SDD, was used to record exit dose. No buildup was added to the MapCHECK2 so the effective depth of measurement was 2 cm, close to *d*_max_. The measured doses were resampled from the 5 mm measurement grid to a 0.38 mm grid so that a gamma analysis could be performed with the same distance‐to‐agreement tolerance as with the other images in this test. These images were imported into SNC Patient software (Sun Nuclear Corporation) for gamma analyses. SNC Patient performed the gamma analyses using 2%/3 mm instead of 2 mm since the MapCHECK2 doses were measured at 150 cm and were not rescaled to 100 cm. The dose from these beams was also calculated using an Eclipse (Varian Medical Systems) treatment planning system v13.6 using the Acuros XB algorithm in a geometry as close as possible to that of the EPID irradiation, using a 20 cm thick water equivalent rectangular phantom, 90 cm SSD, and a 6 cm water equivalent slab, 38.5 cm away from the thicker phantom, representing the EPID (148.5 cm SSD). The dose in a plane 1.5 cm deep in the thinner slab (150 cm from the source) was calculated for each pair of fields, the original and that with induced errors. These dose grids were calculated from a 3D dose grid with a 2 mm resolution, resampled to 0.39 mm resolution and exported to the SNC Patient software to perform the gamma analyses using 2%/3 mm since the calculation plane was 150 cm from the source and not scaled back to 100 cm. Thus, the effective depth of measurement for all three analyses was close to *d*_max_, the beams passed through the same 20 cm thick solid water slab, the source and phantom to detector or calculation plane was the same, the gamma analysis parameters were the same for all images when scaled back to 100 cm distance, and the dose grid resolutions were nearly the same. The gamma passing rates for each field for the Eclipse calculations, MapCHECK2 measurements, and the PerFRACTION measurements were compared.

#### 2.C.9 Head phantom setup errors

An anthropomorphic head phantom was used to mimic a patient receiving radiation treatment. It contains realistic heterogeneities which will cause dose gradients in the EPID image. Depending on the magnitude of the phantom shift relative to the baseline image and the gamma tolerances set, these gradients may cause PerFRACTION to generate gamma failures. An even larger affect will be caused by shifts of the air–tissue interface in the phantom. The head phantom was treated with a 9 cm × 11.5 cm, anterior–posterior (AP) open field on our TrueBeam machine. In the first fraction, the head phantom was treated according to the plan, and an EPID image was captured and used as the baseline image. The head phantom was then imaged with induced errors in lateral and longitudinal shifts and/or roll rotations. The shift error was induced by moving the couch by predetermined distances using the digital readout from the in‐room monitor. The rotational error was induced by adjusting one of the three screws of a leveling plate that supports the phantom. A digital level was used to set the angle of the leveling plate. The following six errors were induced to the setup of the head phantom: (1) 1 mm lateral and longitudinal shifts; (2) 2 mm lateral and longitudinal shifts; (3) 0.5° roll rotation; (4) 1.0° roll rotation; (5) 1 mm lateral and longitudinal shifts plus 0.5° roll rotation; (6) 2 mm lateral and longitudinal shifts plus 1° roll rotation. These errors were selected to range from extremely small to those that are routinely seen during a treatment course. Each error was induced in a separate treatment fraction and an EPID image was captured for each scenario. An open field rather than a modulated field was chosen to not obscure the relatively small changes in each EPID image caused by the various induced positional shifts. In PerFRACTION, the EPID images were compared against baseline using Gamma analyses. We performed PerFRACTION analyses using gamma criteria of 1%/1 mm, 2%/2 mm and 3%/3 mm and recorded the gamma passing rates for each induced error.

Table 1 summarizes the errors we manually induced and the errors expected in EPID images. Because the EPID was positioned at SDD of 150 cm and the images were not scaled to 100 cm SDD for certain tests, the geometric errors were magnified by 1.5 times the nominal values.

**Table 1:** Items and induced errors that were tested in this work

Tested items	Induced errors (defined at isocenter)	Expected errors in EPID images (SDD = 150 cm)
Jaw position	1, 2, 3, 4 mm	1.5, 3, 4.5, 6 mm
MLC position	1, 2, 3, 4, 5 mm	1.5, 3, 4.5, 6, 7.5 mm
Collimator rotation	1, 2, 3°	1.3, 2.6, 3.9 mm^a^
Linac output	0.5%, 1.0%, 1.5%	0.5%, 1.0%, 1.5%
Couch position	1, 2, 3 mm	1.5, 3, 4.5 mm
Rails position with delivery of an open field	Rails in vs. out	Changes in EPID‐measured image
Rails position with delivery of a VMAT field	Rails in vs. out	Changes in EPID‐measured image
MLC position in IMRT fields (field 1, 2, 3)	Gamma passing rates of 93.1% 84.0% and 89.9% with 2%/2 mm gamma criteria using MC2	Gamma passing rates within 3% of MC2 values
Head phantom setup	0.5° roll 1 mm shifts 1.0° roll 1 mm shifts, 0.5° roll 2 mm shifts 2 mm shifts, 1 deg roll	All errors can be detected

a The values represent the maximum deviation of the jaw after the collimator rotates 1, 2, or 3°.

### 2.4 Constancy check of treatment delivery and EPID imager combined

Because we were looking for small changes between baseline and subsequent error‐induced fields, the constancy of the linac and EPID panel sensitivity was important and over the time of our study, was measured. The delivery of an open field and the measurement of integrated exit dose images were repeated four times before each session of study measurements. During each delivery, the field was moded‐up from retracted positions of the jaws and MLCs. These images were analyzed using PerFRACTION with DD analyses.

## 3 RESULTS

### 3.A Jaw position errors

The DTA tolerance was decreased as described in the Methods section until failing pixels were seen. The smallest jaw position shift, which is 1.5 mm in the EPID image, was apparent with the DTA tolerance set to 1.3 mm. Fig. 1 shows that PerFRACTION can detect the induced error area in the EPID image where the jaw positions changed from nominal position. The sensitivity of PerFRACTION in identifying a jaw position error is 0.2 mm.

### 3.B MLC position error

The smallest leaf position error, which is 1.5 mm in the EPID image, became apparent, appearing yellow or orange in the color map when the DTA tolerance was set to 1.1 mm. Fig. 2 shows that the PerFRACTION DTA analysis marks the area where the MLC positions changed from the nominal position. The analysis of this test using the 20 cm thick solid water phantom showed a sensitivity of 0.45 mm which is within 0.1 mm of the test without phantom. The sensitivity of PerFRACTION in identifying a MLC leaf position error is on average 0.4 mm.

### 3.C Collimator rotation error

Fig. 3 shows that for collimator rotational error of 1° (which corresponds to a jaw position shift up of 1.3 mm in the EPID image). PerFRACTION DTA analysis began to demonstrate the error when the DTA tolerance was set to 0.9 mm which is equivalent to a 0.7° collimator rotation. For induced collimator rotational errors of 2 and 3°, PerFRACTION DTA analyses began to display failing pixels when the DTA tolerance was equivalent to 1.7 and 2.5° respectively. The sensitivity of PerFRACTION in identifying a collimator rotation error is therefore 0.5°.

### 3.D Machine output error

For induced errors of 0.5%, 1.0%, and 1.5%, PerFRACTION DD showed failing pixels when the DD tolerance was set to 0.5%, 1.2%, and 1.6%, respectively. The sensitivity of PerFRACTION to identify an output error is 0.2%.

### 3.E Couch position errors

For the induced couch position shift, which was at a minimum 1.5 mm in the EPID image, PerFRACTION began to display failing pixels when the DTA tolerance was set to 1.7 mm. The sensitivity of PerFRACTION in identifying a couch position error is therefore 0.2 mm.

### 3.F Rail position error with an open field

**Figure 4:**
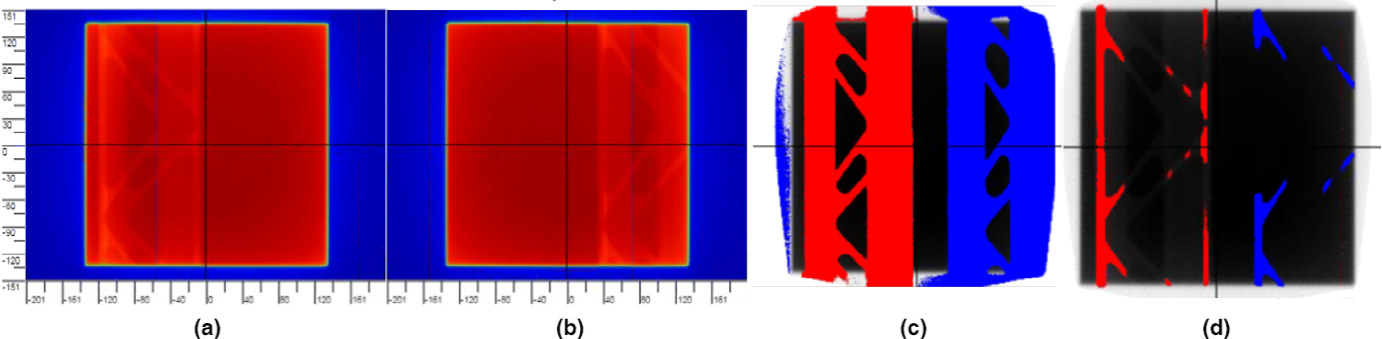
Rail position error test with the delivery of a static field. (a) EPID‐measured baseline image with the rail at the out‐most position under the couch. (b) EPID‐measured image with induced rail position error, in which the rail was moved to the center of the couch. (c) Image comparison using DD method with a tolerance of 1%. The pixels are rendered red or blue if it is too hot or too cold compared to the baseline. (d) Image comparison using DD method with a tolerance of 8%.

Fig. 4(a,b) shows the changes in the integrated image due to rail position error with a static field. When the DD tolerance is set to any value up to 8%, PerFRACTION renders blue and red pixels in the color map (Fig. 4c,d), meaning the maximum DD is about 8%. This test indicates that PerFRACTION can identify a rail position error with an open static field irradiation, and in this case represented a dose error up to 8%.

### 3.G Rail position error with a VMAT field

**Figure 5:**
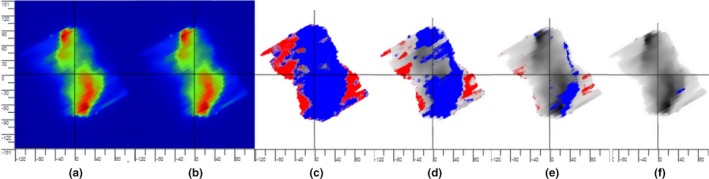
Rail position error test with the delivery of a VMAT field. (a) EPID‐measured baseline image with the rail at the out‐most position under the couch. (b) EPID‐measured image with induced rail position error, in which the rail was moved to the center of the couch. (c) Image comparison using DD method with a tolerance of 0.2%. The pixels are rendered red or blue if it is too hot or too cold compared to the baseline. (d) DD analyses with a tolerance of 1%. (e) DD analyses with a tolerance of 2%. (f) DD analyses with a tolerance of 3%.

Fig. 5(a,b) display the integrated exit dose images with rails in or out with a VMAT field irradiation. The difference between Fig. 5(a,b) is not visually obvious but can be quantitated by PerFRACTION. As shown in Fig. 5(c–f), PerFRACTION displays a few blue or red pixels with DD tolerance set to 3%, and displays more failing pixels with reduced DD tolerances. This test indicates that PerFRACTION is sensitive in identifying a rail position error during VMAT delivery, with as much as a 3% dose error being uncovered in this case.

### 3.H MLC errors in IMRT fields

**Figure 6:**
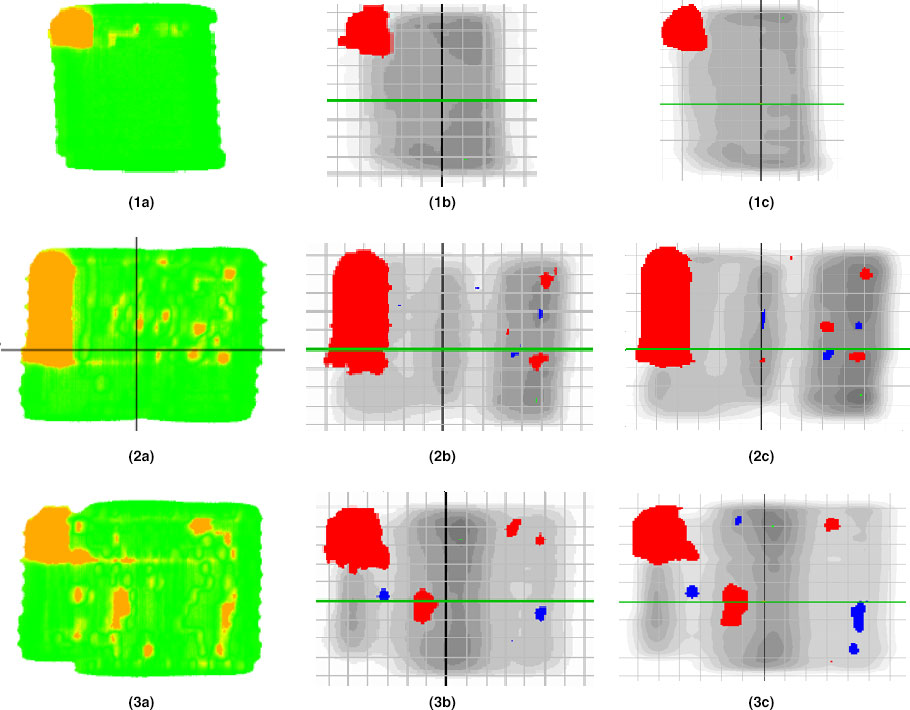
MLC shift error in IMRT fields with a 20 cm thick phantom in the beam path. 1a, 2a, 3a display the PerFRACTION analyses of EPID measurements of baseline fields and fields with induced MLC shift error. 1b, 2b, 3b display the SNC Patient analyses of MapCHECK2 measurements of the same baseline fields and the same test fields. 1c, 2c, 3c display the SNC Patient analyses of Eclipse‐calculated dose of the same baseline fields and the same test fields. All three tests are with the same irradiation geometry.

Fig. 6 shows that PerFRACTION's gamma analysis using the EPID images identified nearly identical failing pixel regions as the SNC Patient software using either MapCheck2 measurements or the Eclipse‐calculated dose images for all three fields. The PerFRACTION‐calculated gamma passing rates using EPID images were within 2.2% of the MapCHECK2 measured fields and the Eclipse‐calculated fields. The PerFRACTION, MapCHECK2, and Eclipse passing rates for the first field were 94.2%, 93.1%, and 93.9%, for the second field, 86.2%, 84.0%, and 85.6%, and for the third field, 90.0%, 89.9%, and 89.8%, respectively.

### 3.I Head phantom setup errors

**Figure 7:**
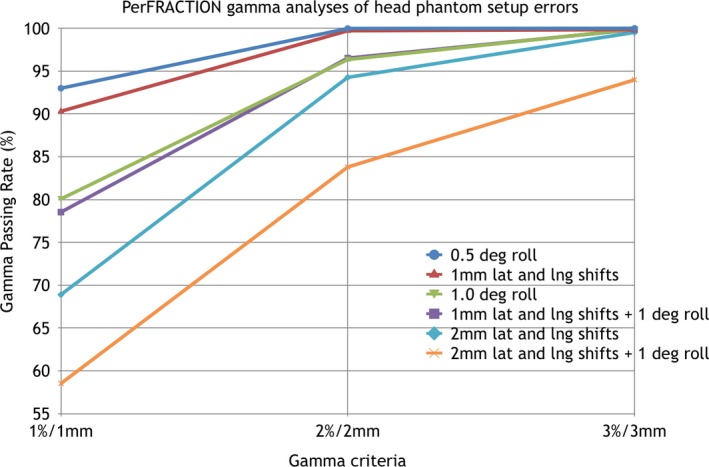
Display of PerFRACTION‐calculated gamma passing rates of each head phantom setup error with respect to gamma criteria of 1%/1 mm, 2%/2 mm, and 3%/3 mm.

**Figure 8:**
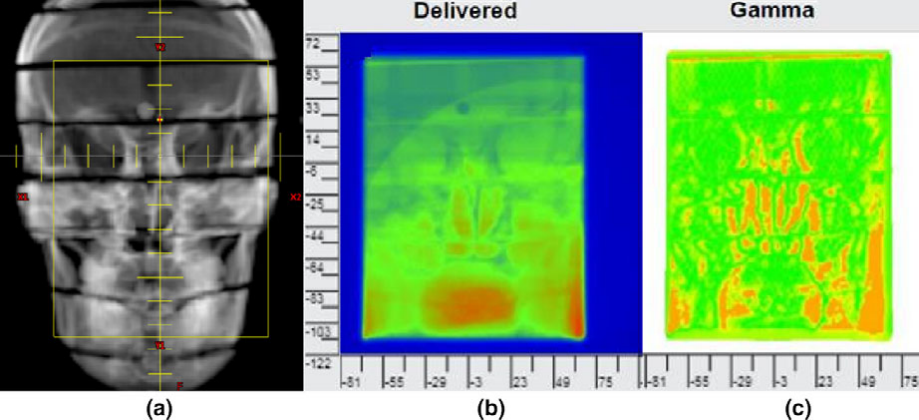
Display of (a) the beams eye view of the AP field superimposed over the DRR, (b) the integrated EPID image acquired during the delivery with 2 mm lateral and longitudinal shifts and 1° roll, and (c) the gamma analysis map of the 2 mm shifts and 1° roll for 2% dose and 2 mm DTA tolerances.

Fig. 7 displays PerFRACTION‐calculated gamma passing rates relative to gamma tolerance criteria for each induced error. When 1%/1 mm gamma criteria was used, gamma passing rates for the 0.5° rotational error, the 1 mm shift error, 1° rotational error, 1 mm shift plus 0.5° rotation error, 2 mm shift error, and 2 mm shift plus 1° rotational error were 93%, 90.3%, 80.1%, 78.5%, 68.9%, and 58.5%, respectively. As the tolerance levels were increased, passing rates successively increased with the passing rate order preserved. Thus, the smallest induced errors can be detected if tight gamma tolerances are selected and a gamma passing rate above 93% is used as the passing criteria. Fig. 8 displays the beams eye view of the AP field superimposed over the Digitally Reconstructed Radiograph (DRR), the corresponding EPID image acquired during the delivery with 2 mm lateral and longitudinal shifts and 1° roll, and the gamma analysis map for this error for 2% dose and 2 mm DTA tolerances.

Table 2 summarizes the sensitivity of PerFRACTION.

**Table 2:** Induced errors, DD, and/or DTA tolerance used, PerFRACTION‐calculated Gamma passing rates, and the sensitivity of PerFRACTION

Tested items	Induced errors	DD and/or DTA tolerance, or Gamma passing rates	Sensitivity of PerFRACTION
Jaw position	1.5 mm	1.3 mm	0.2 mm
MLC position	1.5 mm	1.1 mm	0.4 mm
Linac output	0.5%, 1.0%, 1.5%	0.5%, 1.2% and 1.6%	0.2%
Collimator rotation	1, 2 and 3°	0.7, 1.7 and 2.5°^a^	0.5°
Couch shift	1.5 mm	1.7 mm	0.2 mm
Rail position change with an open field	Rails out vs. rails in	≤8%	Incorrect rail position detected
Rail position change with a VMAT field	Rails out vs. rails in	≤3%	Incorrect rail position detected
MLC position in IMRT fields (field 1, 2, 3)	Gamma passing rates of 93.1% 84.0% and 89.9%	94.2%, 86.2%, 90.0%	2.2% max
Head phantom setup	0.5° roll 1 mm shifts 1.0° roll 1 mm shifts, 0.5° roll 2 mm shifts 2 mm shifts, 1° roll	≤1%/1 mm ≤1%/1 mm ≤1%/1 mm ≤1%/1 mm ≤2%/2 mm ≤3%/3 mm	0.5° roll or 1 mm shifts

a DTA method was used to analyze the positional change of the jaw, which is correlated with a collimator angle.

### 3.J Constancy check

The constancy check showed that the EPID‐measured integrated exit dose images were consistent with a deviation of 0.2% or less in dose. With this small error in reproducibility of the linac output, we made no corrections for this effect.

## 4 DISCUSSION

Interpretation of exit dosimetry results depends on the sensitivity of the system to detect an error. Ideally, the detection system would be able to discern errors much smaller than are clinically relevant so even small errors can be accurately identified. The software allows the user to choose the level of sensitivity for clinical use depending on departmental policy. Because this system is fully automated so that no physicist time is required for data acquisition and evaluation, daily patient treatment QA is feasible. In this study, we introduced a range of small known errors in either geometric or dosimetric parameters and measured the ability of PerFRACTION to detect them. The induced errors were designed to simulate those that might be encountered over the course of typical treatments, such as changes in the patient anatomy, changes in the portion of the beam passing through couch and immobilization structures, changes in patient position, and changes in beam parameters. For example, the test for detection of changes in MU would simulate dose changes received by the EPID if the patient thickness changed. At the PerFRACTION sensitivity level for changes in dose of 0.2%, there is the ability to detect a <1 mm change in thickness.

PerFRACTION may be less sensitive to a vertical couch error. For an AP or PA beam, then the degree of error found by PerFRACTION will be related to the magnification of the irradiated region of the image. For a 1 cm vertical couch error and no other errors, a 10 cm diameter‐centered dose region will appear to be 1 mm larger or smaller in diameter than for the correct (baseline) vertical couch position. This will be detected as a lateral shift. A 4 mm vertical couch error will cause PerFRACTION to detect a 0.2 mm lateral shift (its limit of detectability) for the pixels on the perimeter of the 10 cm dose region. For fields at an angle to the vertical, a vertical couch error would result in a shift on the EPID whose magnitude corresponds to the sine function of the angle. For example, for a lateral beam and a 3 mm vertical couch error, the entire 3 mm error would be represented in the image.

We found that PerFRACTION can resolve the geometric error caused by jaw or MLC leaf within 0.4 mm. It can determine the dose change in an EPID image within 0.2%. It can determine a collimator rotation error within 0.5° (based on the corresponding distance error). PerFRACTION can alert the user to the misplacement of the couch rail by either a static field or a VMAT field.

Although the most common 2D image analysis method is the gamma analysis with both a dose and DTA tolerance, in this study we wanted to be more exacting in the partition of the source of error found. In this work, to better understand the sensitivity of the system in analyzing a dosimetric error we used the DD method without DTA because DD directly analyzes dosages. Similarly, for geometric error we used the gamma method without a dose tolerance because it directly analyzes geometric errors such as jaw or MLC shifts.

The IMRT field tests provide a realistic measure of the system's sensitivity to the composite of MLC position errors that could occur in a clinical IMRT plan. The three‐way gamma passing rate test results were nearly the same for the three fields tested. However, the tests were not perfect in that the Eclipse simulation of the EPID geometry, as close as it was to representing that of the EPID, is not exact, and the Eclipse‐calculated dose maps are not exactly comparable to the exit dose images measured by the EPID. Also, the MapCHECK2 doses were measured with a much coarser grid of 5 mm compared to the submillimeter EPID and Eclipse results. These differences in part, may be contributing to the up to 2.2% difference in gamma passing rates found in the three‐way comparison.

We induced very small to moderate lateral and longitudinal shifts and rotational errors during the setup of a head phantom to mimic realistic clinical situations. Because the anatomy of the head phantom contains heterogeneous tissues such as soft tissue, air, and bone, when the head phantom was shifted from baseline position, the pattern of the exit dose image changed. PerFRACTION gamma analysis was able to demonstrate even the smallest induced errors if correspondingly tight tolerance levels were used in the analysis, demonstrating that PerFRACTION has the sensitivity to be able to alert the user to errors that are even smaller than might be considered clinically significant. Actual patient results using PerFRACTION will be reported in a separate publication.

Acquiring EPID images for PerFRACTION is limited to couch‐gantry angle combinations that don't cause imager to couch or patient collisions. For coplanar beams, 150 cm source‐imager distance allows imaging even for most off center couch positions. For noncoplanar beams, increasing the source‐imager distance from 150 to 170 cm greatly increases the range of beams that can be imaged, but there will still be those that could cause a collision. In our experience, for a typical 10 beam noncoplanar plan, at most two beams cannot be safely imaged.

## 5 CONCLUSIONS

The PerFRACTION system, which is comprised of software that automatically retrieves EPID images for each fraction and compares them to the baseline, typically the first fraction, is sensitive enough to provide useful and actionable information about the reproducibility of treatment delivery and patient setup. This type of fully automated daily patient treatment QA using the ubiquitous EPID, is feasible since it uses virtually no physicist's time and fills an important unmet need for a better understanding of the accuracy of daily treatment.

## CONFLICT OF INTEREST

Dr. Arthur Olch receives research funding from Sun Nuclear Corporation but no funding for this work was provided.
